# Elucidation of disease etiology by trans-layer omics analysis

**DOI:** 10.1186/s41232-021-00155-w

**Published:** 2021-02-08

**Authors:** Yuya Shirai, Yukinori Okada

**Affiliations:** 1 Department of Statistical Genetics, Osaka University Graduate School of Medicine, 2-2 Yamadaoka, Suita, Osaka, 565-0871 Japan; 2 Department of Respiratory Medicine and Clinical Immunology, Osaka University Graduate School of Medicine, Suita, 565-0871 Japan; 3 Laboratory of Statistical Immunology, Immunology Frontier Research Center (WPI-IFReC), Osaka University, Suita, 565-0871 Japan

## Abstract

To date, genome-wide association studies (GWASs) have successfully identified thousands of associations between genetic polymorphisms and human traits. However, the pathways between the associated genotype and phenotype are often poorly understood. The transcriptome, proteome, and metabolome, the omics, are positioned along the pathway and can provide useful information to translate from genotype to phenotype. This review shows useful data resources for connecting each omics and describes how they are combined into a cohesive analysis. Quantitative trait loci (QTL) are useful information for connecting the genome and other omics. QTL represent how much genetic variants have effects on other omics and give us clues to how GWAS risk SNPs affect biological mechanisms. Integration of each omics provides a robust analytical framework for estimating disease causality, discovering drug targets, and identifying disease-associated tissues. Technological advances and the rise of consortia and biobanks have facilitated the analyses of unprecedented data, improving both the quality and quantity of research. Proficient management of these valuable datasets allows discovering novel insights into the genetic background and etiology of complex human diseases and contributing to personalized medicine.

## Background

**Fig. 1:**
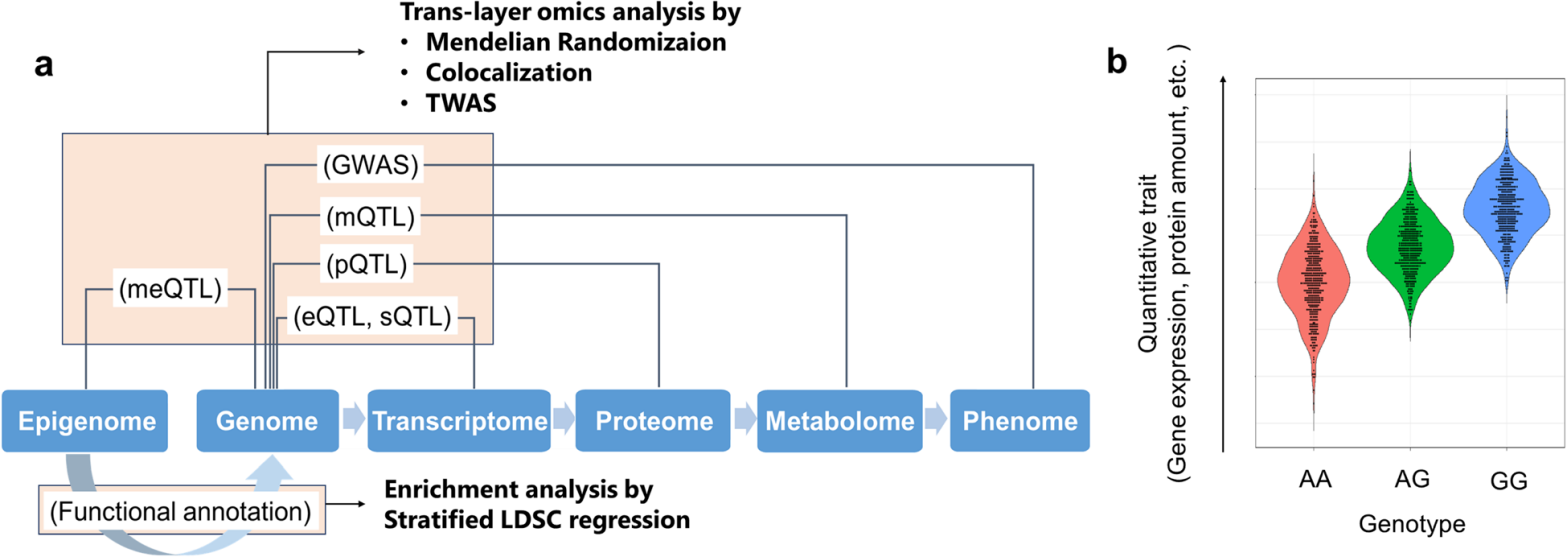
Overview of trans-omics layer analysis. **a** Omics data constituting each layer of the central dogma and analysis methods that combine them. GWASs show associations between genotype and phenotype. Quantitative trait loci (QTL), which reflect how much genetic variants affect data in other layers, can link genome to other omics. Epigenetic data provide functional genomic annotations based on various experiments. These data connecting each layer are described in red squares and analysis methods that integrate them are shown in bold. GWAS: genome-wide association study, TWAS: transcriptome-wide association study, eQTL: metabolite QTL, sQTL: splicing QTL, pQTL: protein QTL, mQTL: metabolite QTL, meQTL: methylation QTL, LDSC regression: Linkage disequilibrium score regression. **b** An example of QTL. The continuous quantitative traits, such as gene expression level and protein amount, are plotted for each genotype

Genome-wide association studies (GWASs) are study designs for evaluating associations between genetic variants and phenotypic traits across the genomes, revealing the genetic impacts on a variety of phenotypes since its first success in 2002 [1–3]. In recent years, the rise of international consortia and biobanks has enabled GWAS’s application on a scale of more than 1 million samples, which can identify a large number of genetic risk variants. However, little is known about how GWAS results should be translated into disease etiology and novel drug discovery. To correctly interpret GWAS results, it is useful to utilize additional information, such as transcriptome, proteome, and metabolome, which are the components of the central dogma (Fig. 1a). Assessment of the translation from genotype to phenotype, including the role of omics, will enable us to understand how genetic diversity impacts our health (trans-layer omics analysis).

Ideally, researchers prefer to use identical samples with data across each omics. However, few datasets contain all the omics data in individuals. While it would be ideal for defining a new sample and collecting all of the omics, genetic and phenotypic data, that undertaking has enormous cost and time required. While individual raw data is often restricted in its use due to ethical issues, de-identified or anonymized analysis results are available in many biological databases. We can perform comprehensive analyses for various phenotypes by integrating the accumulated data with our own data. In particular, the quantitative trait loci (QTL), or loci associated with a variation of a quantitative trait (Fig. 1b), play an essential role in trans-layer omics analysis.

This review introduces several useful resources that can be applied to trans-layer omics analysis and describes how the data can be utilized, mainly focusing on QTL.

### Transcriptome

More than 90% of the risk variants identified by GWASs have been found in non-coding regions [4, 5], and as a result, rarely alter the amino acid sequence. Several studies have reported that many GWAS risk variants in the non-coding region are enriched in regulatory regions involved in gene transcription, including enhancers and promoters [6, 7]. These findings motivated the investigation of genetic variants, called expression quantitative trait loci (eQTL), that affect gene expression levels. Researchers have identified numerous eQTL, some of which are available in several curated databases (Table 1). The effects of eQTL are tissue- and cell-specific [8, 18], and it is important to use eQTL data derived from the appropriate tissues and cells in relation to the target phenotype.

**Table 1:** Useful resources and databases related to QTL

Name	Data	Description	URL	Reference
**GTEx**	**eQTL, sQTL**	**The project examining 15,201 RNA-sequencing samples from 49 tissues and whole-genome sequencing of 838 donors.**	https://gtexportal.org/home/	[8, 9]
**DICE**	**eQTL**	**The resource of eQTL considering all human immune cell types.**	https://dice-database.org/	[10]
**Immugen**	**eQTL**	**The resource of eQTL neutrophils and splenic CD4+ T cells across a panel of 40 mouse inbred strains.**	http://www.immgen.org/	[11]
**eQTLgen**	**eQTL**	**The consortium which incorporates 37 eQTL datasets for the blood from 31,684 individuals.**	https://www.eqtlgen.org/	[12]
**PsychENCODE**	**eQTL**	**The consortium which generates a comprehensive online resource for the adult brain across 1866 individuals.**	http://resource.psychencode.org/	[13]
**eQTL Catalogue**	**eQTL**	**The database which contains quality controlled, uniformly re-computed QTLs from 19 eQTL publications.**	https://www.ebi.ac.uk/eqtl/	[14]
**SomaLogic serum pQTL**	**pQTL**	**The serum pQTL summary statistics form 3301 European healthy samples (INTERVAL study).**	http://www.phpc.cam.ac.uk/ceu/proteins/	[15]
**mQTLdb**	**metylation QTL**	**The methylation QTL summary statistics of 1000 mother-child pairs at serial time points across the life-course (ARIES).**	http://www.mqtldb.org/	[16]
**QTLbase**	**Various QTLs**	**The database compiling genome-wide QTL summary statistics for human molecular traits across > 70 tissue/cell types.**	http://mulinlab.org/qtlbase	[17]

The Genotype-Tissue Expression (GTEx) Project has built a large-scale database of organ-specific eQTL data. The latest GTEx database is version 8, deployed in 2020, which includes curated data on as many as 15,201 RNA sequencing samples from 49 tissues of 838 donors [9]. In addition to eQTL information, the GTEx also contains genetic variants, splicing quantitative trait loci (sQTL), affecting gene splicing. The sQTL data allow us to develop hypotheses about genetic polymorphisms in splicing, leading to diversity in the RNA transcribed from a single gene.

Several eQTL analyses for individual blood cell types have been successfully performed due to easy access to the sample and the cell diversity [10, 19–21]. In these studies, cell types were clustered by sorting in the laboratory or estimation in silico.

The DICE (database of immune cell expression, expression quantitative trait loci (eQTL) and epigenomics) project was established to define the transcriptional and epigenomic landscape of many human immune cell types in relation to genetic variation [10]. As the first report from this project, Schmiedel et al. performed the eQTL analysis for 13 primary immune cell types (three innate immune cell types, four naive adaptive immune cell types, and six CD4^+^ T memory cell types) and two activated cell types isolated from 106 leukapheresis samples provided by 91 healthy subjects in the San Diego area. They identified a total of 12,254 genes with cis-eQTL and a large fraction (41%) of these genes showed a strong cis-association with genotype only in a single cell type. Furthermore, they confirmed several cases in which the cell types with eQTL corresponding to GWAS risk SNPs related to the well-known pathogenesis.

As for the eQTL analysis of non-Europeans, Ishigaki et al. conducted the eQTL analysis on five immune cell types (CD4+ T cells, CD8+ T cells, B cells, natural killer cells, and monocytes) and unfractionated peripheral blood from 105 healthy Japanese volunteers [21]. In this study, gene expression levels were predicted from individual genotype data based on the developed eQTL dataset and public epigenetic data. Subsequently, the association analysis between the estimated gene expression levels and 15 diseases state was performed. Finally, the cell-specific pathway activity was predicted by integrating the direction of eQTL effects. This framework applied to rheumatoid arthritis (RA) revealed that activation of the TNF pathway in CD4+ T cells plays a vital role in RA etiology.

Association analyses for genome-wide gene expression, as was done in this study, are called transcriptome-wide association studies (TWASs). TWASs use penalized regression techniques, such as LASSO, Ridge, or Elastic net, which incorporate eQTL reference data as training data and evaluate associations between predicted gene expression levels and a target trait. TWAS can also be performed with only GWAS summary statistics and external eQTL reference data [22, 23]. However, we should carefully interpret TWAS results because non-causal genes co-regulated with causal genes are likely significant, resulting in TWAS results’ bias. Fine-mapping of causal gene sets deals with this problem by incorporating linkage disequilibrium data and provides less unbiased results [24, 25].

Most transcriptome studies focus on coding genes because many non-coding genes have unknown functions, and the analysis results can be challenging to interpret. Sakaue et al. developed a method for estimating GWAS-target miRNAs and GWAS-related tissues by integrating GWAS summary statistics and miRNA expression data [26]. Analyses incorporating the transcriptome are expected to continue developing with increasing resources and maturating analytic techniques for non-coding gene data.

### Single-cell analysis

Single-cell RNA sequencing makes it possible to evaluate gene expression at an unprecedentedly fine resolution in individual cell types and identify rare populations without assumptions. The previous classification of cells by surface markers could only identify existing cell types and had difficulty identifying heterogeneity in captured cell types [27]. Single-cell RNA sequencing is currently evolving with respect to technology and sample size. Monique et al. conducted eQTL analysis using single-cell RNA sequencing to identify eQTL for rare populations and reported variants that alter gene co-expression [28]. The eQTL analysis for single-cell RNA sequencing can analyze individual cell types and freely definable clusters such as cell lineages, providing a more flexible analysis framework. More and more cell-type-specific and cell-cluster-specific eQTL will be identified in the near future [29].

In general, eQTL analyses that handle RNA data have a limit in that they capture a snapshot at a single point in time, not reflective of transcriptome fluctuations. Therefore, many eQTL are conditional, and some can only be identified through cell activation or cell differentiation [30, 31]. Davenport et al. focused on transcriptome changes associated with drug administration [32]. They reported how much the eQTL were impacted by IL6 antibody treatment in SLE patients, resulting in expression changes. This study showed that the eQTL analysis using RNA-seq data at multiple times (at 0, 12, and 24 weeks of anti-IL6 drug administration) increased the number of identifiable eQTL compared to the analysis from one point in time. This study revealed that several eQTL effects were enhanced at a high total IFN level or by IL6 antibody administration, and each of the eQTL was enriched in ISRE motif and IRF4 motif. These findings suggested that these transcription factors (TFs) binding motifs may be key regulatory mediators of environmental stimuli and potential therapeutic targets [33].

Mass cytometry is another single-cell modality and provides different cell-type-specific profiles. Mass cytometry measures a limited number (~ 40) of pre-selected markers, but these markers are supported by decades of experimental evidence that they are useful for defining cellular heterogeneity [34]. Zhang et al. defined stromal and immune cell populations overabundant in RA joint synovial tissues by integrating single-cell RNA sequencing and mass cytometry data [35]. They found that several specific immune cells, categorized by genetic and proteomic profiles (e.g., THY1^+^HLA-DRA^hi^ sublining fibroblasts), were increased in RA synovium. This study showed that the integration of multiple experimental modalities helps us select trait-specific cells by increasing the individual cells’ information. QTL analyses of these cells central to the etiology should provide more evident and profound insights into genetic impacts on various diseases.

### Proteome

The proteome comprises the entire protein complement produced in an organism or system. Sun et al. investigated the associations between genetic variants and 2994 proteins in 3301 Europeans [15]. They identified 1927 genetic loci, which altered the plasma protein amount between 1478 proteins and 764 genomic regions, known as protein quantitative trait loci (pQTL). Of note, only 10–20% of the previously reported cis eQTL were cis pQTL, while cis pQTL were significantly enriched in eQTL for the corresponding gene. Comparison between eQTL and pQTL studies may be influenced by differences in sample size, tissues, and technology platforms used in each analysis. Nonetheless, this study suggested that genetic effects on plasma protein abundance are often, but not exclusively, driven by regulation of mRNA.

Plasma proteins play essential roles through biological processes and represent a significant resource for drug targets [36]. Mendelian randomization (MR) is a useful method for exploring diseases-causing proteins, which can be therapeutic targets. Instead of allocating interventions or non-intervention as in randomized controlled trials (RCT), MR allocates subjects according to risk variants of the causative traits [37]. MR is an attractive method for conducting RCT-like research despite being feasible with existing data. MR studies can be performed if the two traits are in different cohorts. Thus, the analysis platform with access to various GWAS results has been currently established [38]. Zheng et al. conducted a large-scale MR analysis of 1002 proteins on 225 traits with five extensive pQTL datasets [39]. They found 111 causal relationships between 65 proteins and 52 disease-related phenotypes. When these relationships were queried against a curated drug database, previously defined associations between approved drugs and their target proteins were more likely to be identified. This finding supported MR as a useful tool to search for drug targets.

### Metabolome

The metabolome consists of small molecules that are intermediates or products of metabolism, ranging from peptides and lipids to drugs and pollutants. Several studies that tested the associations between genetic variants and serum metabolites have reported hundreds of loci changing serum metabolite amount, or metabolite quantitative trait loci (mQTL), which were often mapped to genes encoding for enzymes or transporters [40–42]. The kidneys play an important role in the regulation of blood metabolite by controlling the amount of urinary metabolites [43]. As a result of excretion, urinary metabolites are more diverse than serum metabolites. Therefore, they can reflect individual differences that cannot be captured in blood metabolites. This fact inspired the investigation of associations between urinary metabolites and genetic variants [44, 45]. Schlosser et al. performed the GWAS for the urinary concentrations of 1172 metabolites among 1627 patients with reduced kidney function [44]. They identified 240 urine metabolite-mQTL associations and found that the loci included 90 unique genes. These genes’ expressions were seen in organs involved in the absorption and metabolism, such as the kidney, liver, and small intestine.

Furthermore, they used the colocalization method to confirm whether a GWAS risk variant was also responsible for mQTL signals in the locus. GWAS and QTL signals can overlap for three reasons: two independent causal variants in linkage disequilibrium (linkage), a single causal variant affecting the GWAS trait via gene expression modulation (causality), or a single causal variant affecting both traits independently (pleiotropy) [46]. Colocalization helps us distinguish causality and pleiotropy from linkage, which is essential for identifying targets that drive GWAS risk loci.

### Epigenome

The epigenome describes a biological phenomenon where chemical compounds can modify or mark the genome to affect gene expressions. DNA methylation at CpG dinucleotides plays an important role in gene regulation by altering DNA affinity with TF or chromatin-binding proteins. DNA methylation can occur for various reasons: normal development such as genomic imprinting, aging, environmental factors, or genetic factors. In these, genetic factors can be inherited and diversify the innate methylation status among individuals. Gaunt et al. evaluated the genetic influences on DNA methylation, or methylation quantitative trait loci (meQTL), in the human blood at five different life stages: children at birth, childhood, adolescence, and their mothers during pregnancy and at middle age [16]. They identified 30,000 significant associations at each time point and revealed that the genetic heritability was highly stable at about 20% throughout the life stages. They also showed that meQTL likely overlapped with eQTL, as reported in GTEx, and enriched in GWAS risk loci for complex diseases, such as Alzheimer’s disease and schizophrenia. Because DNA methylation is generally involved in gene regulation, genetic variants that affect methylation status can also affect gene expression levels. Their result supported that some GWAS risk variants may impact our health by altering gene expression via methylation.

While QTL are beneficial information in evaluating the effects of GWAS risk SNPs, most QTL analyses do not cover all tissues and probably have insufficient power in some tissues due to a lack of adequate sample size. It is useful to consider what functional annotations target variants are located in (Table 2). ENCODE [47], ROADMAP Epigenomics [49], and BLUEPRINT [50] have accumulated considerable resources, mapping regulatory annotations in the genome by profiling chromatin functions, including DNase hypersensitivity sites, several types of histone markers, and the binding sites of chromatin-related proteins in many cells and tissues. ENCODE, which aims to catalog all functional elements of humans and mice, was updated in 2020 by expanding the target cell types and tissues and adding new annotations, including RNA-binding protein regions and chromatin loops [48]. ENCODE, version 3, integrates a vast amount of accumulated data into novel annotations for 926,535 candidate cis-regulatory elements (cCRE), covering 7.9% of the human genome and 339,815 cCRE covering 3.4% of the mouse genome. The registered data accumulated in several large consortia including ENCODE is tremendous; a tool for efficiently searching them has also been released [52].

**Table 2:** Useful resources and tools related to epigenetic data

Name	Description	URL	References
**ENCODE**	**The project which aims to catalog all functional elements genome of humans and mice by various assays.**	https://www.encodeproject.org/	[47, 48]
**Roadmap Epigenomics**	**The project which aims to produce a public resource of human epigenomic data.**	http://www.roadmapepigenomics.org/	[49]
**BLUEPRINT**	**The European project which aims to generate epigenomic maps of blood cells.**	https://www.blueprint-epigenome.eu	[50]
**DEEP**	**The German projects which aim to map and functionally interpret reference epigenomes in normal and diseased states.**	http://www.deutsches-epigenom-programm.de	[51]
**DeepBlue**	**The data server which provides epigenetic data collection including data from the above four projects.**	https://deepblue.mpi-inf.mpg.de	[52]
**FANTOM**	**The consortium to assign functional annotations to the full-length cDNAs collected during the Mouse Encyclopedia Project at RIKEN.**	https://fantom.gsc.riken.jp/	[53]
**CHIP-ATLAS**	**The database for visualizing and making use of public ChIP-seq data submitted to the SRA (Sequence Read Archives).**	https://chip-atlas.org/	[54]
**HaploReg**	**The tool to annotate haplotype blocks containing target genetic variants with epigenetic data.**	https://pubs.broadinstitute.org/mammals/haploreg/haploreg.php	[55]
**RegulomeDB**	**The tool to annotate genome regions with epigenetic and eQTL data and classify them by function.**	https://regulomedb.org/regulome-search/	[56]

The FANTOM Consortium has used a unique technique called cap analysis of gene expression (CAGE) to identify promoters and enhancers across hundreds of cells and tissues [53]. Hirabayashi et al. developed NET-CAGE (native elongating transcript), which enables the detection of TSSs of nascent RNAs and quantifies true transcriptional activities of promoters and enhancers at high nucleotide resolution in diverse cell types as well as frozen cells and tissues [57].

Linkage disequilibrium score regression (LDSC regression) is an effective tool for combining GWAS summary statistics with these genome annotations. In the polygenic traits, the *χ*^2^ association statistic for a given SNP in GWASs includes the effects of all SNPs tagged by this SNP [58]. The LD score is calculated by the sum of the linkage disequilibrium *r*^2^ measures of the target variant and the surrounding variants (e.g., variants in a window size of 1 cM around the target variant). The regression of the *χ*^2^ statistics by the LD score in a genome-wide manner provides an estimate of heritability. The modified form, or stratified LDSC regression, partitions SNP heritability by functional genomic annotations and tests whether the GWAS statistics is enriched in the annotations [59]. For stratified LDSC regression, the authors have developed 10 cell-group-specific annotations and 220 cell-type-specific annotations for histone modifications (H3K4me1, H3K4me3, H3K9ac, and H3K27ac) created from ROADMAP Epigenomics data. These annotation sets are useful to implement GWAS enrichment analyses in various tissues [60].

Ishigaki et al. estimated TF enrichment in various diseases using the annotation of TF binding sites defined by 2868 publicly available chromatin immunoprecipitation sequencing datasets for 410 unique TFs [61]. They identified 378 significant enrichments across nine diseases (e.g., NF-κB for immune-related diseases) and revealed that TF clusters characterized based on LD score included TF components which showed similar disease enrichment. LDSC regression provides a flexible framework because it can integrate GWAS summary statistics and customized genome annotations. In the future, functional annotations will continue their significant growth and will be available for genome annotation projects. Enrichment analyses combining these valuable annotations and GWAS data should contribute to the elucidation of complex human traits.

## Conclusions

The available omics data has improved in both quantity and quality. State-of-the-art technology, such as whole-genome sequencing, long lead sequencing, mass cytometry, and single-cell RNA sequencing, should illuminate currently unreachable areas. Furthermore, trans-layer omics analysis empowers the information from the individual omics. If the relationship between the genome and each omics is revealed, various living organisms’ phenomena could be predicted from genome data. The tools introduced in this review are not limited to one type of omics pair but can be applied to various omics data combinations (e.g., colocalization between GWAS and sQTL). The trans-layer omics analyses using these sophisticated methods and a vast amount of data provide novel insights into the genetic background, etiology of complex diseases, and drug discovery, which should contribute to the implementation of personalized medicine.

## Data Availability

Not applicable

## Abbreviations

GWAS: Genome-wide association study
QTL: Quantitative trait loci
eQTL: Expression quantitative trait loci
GTEx: Genotype-tissue expression
DICE: Database of immune cell expression, expression of quantitative trait loci and epigenomics
RA: Rheumatoid arthritis
TWAS: Transcriptome-wide association study
TF: Transcription factor
pQTL: Protein quantitative trait loci
MR: Mendelian randomization
RCT: Randomized controlled trials
mQTL: Metabolite quantitative trait loci
meQTL: Methylation quantitative trait loci
cCRE: Candidate cis-regulatory elements
CAGE: Cap analysis of gene expression
LDSC: Linkage disequilibrium score
